# A Successful Experience of Individualized Vancomycin Dosing in Critically Ill Patients by Using a Loading Dose and Maintenance Dose

**DOI:** 10.3390/ph18050677

**Published:** 2025-05-02

**Authors:** Jorge S. Amador, Álvaro Vega, Patricio Araos, Luis A. Quiñones, Cristián A. Amador

**Affiliations:** 1 Intensive Care Unit, San Borja Arriarán Clinical Hospital, Santiago 8360160, Chile; jamador@uc.cl; 2 School of Chemistry and Pharmacy, Faculty of Medicine, Universidad Andrés Bello, Santiago 8320000, Chile; 3 CleanDrugs®, Hospitales y Atención Sanitaria, Concepción 4030000, Chile; alvarovegac@gmail.com; 4 Hypertension and Kidney Immunology Laboratory, Institute of Biomedical Science, Universidad Autónoma de Chile, Santiago 8910060, Chile; patricio.araos@uautonoma.cl; 5 Laboratory of Chemical Carcinogenesis and Pharmacogenetics, Department of Basic-Clinical Oncology, Faculty of Medicine, Universidad de Chile, Santiago 8500000, Chile; lquinone@uchile.cl; 6 Department of Technology and Pharmaceutical Sciences, Faculty of Chemical and Pharmaceutical Sciences, University of Chile, Santiago 8380000, Chile; 7 Latin American Network for Implementation and Validation of Clinical Pharmacogenomics Guidelines (RELIVAF), Santiago 8500000, Chile; 8 Faculty of Medicine and Science, Universidad San Sebastián, Santiago 7510157, Chile

**Keywords:** vancomycin dosing, critically ill patients, pharmacokinetic model

## Abstract

**Background/Objective:** Vancomycin, a hydrophilic glycopeptide antibiotic with bactericidal activity against Gram-positive microorganisms, is one of the most commonly used antibiotics un the intensive care unit (ICU). Different efforts have been made to achieve a therapeutically effective plasma concentration of vancomycin by using loading and subsequent maintenance doses on an individual basis, but this remains subject to debate. Our objective was to individualize a dosage regimen in a Chilean ICU to optimize the pharmacological treatment of vancomycin by using a population pharmacokinetic model. **Methods**: A quantitative descriptive study was carried out in 51 patients at the adult ICU, San Borja Arriarán Clinical Hospital in Santiago, Chile. The dose of vancomycin was calculated by using a population pharmacokinetic software, the Antibiotic Kinetics^®^, and was subsequently validated with plasma trough levels of the drug through a patient sample. **Results**: The most commonly prescribed loading dose was 1500 mg and the most commonly used maintenance dose was 1000 mg, three times a day. The measured blood plasma concentrations of each patient (16.98 ± 5.423 μg/mL) were compared with the concentrations calculated through the population pharmacokinetic model (14.33 ± 4.630 μg/mL, *p* < 0.05). In addition, a correlation was found between the software-calculated trough concentration versus the measured trough concentration for vancomycin, with a positive correlation between both variables established (R^2^ = 0.65; *p* < 0.0001). No renal side effects were observed in the treated patient group. **Conclusions**: In the present study, a vancomycin dosing model for critically ill patients, based on a population pharmacokinetic model, was successfully implemented for routine clinical practice.

## 1. Introduction

Vancomycin is a hydrophilic antibiotic belonging to the hydrophilic glycopeptide family [1], with bactericidal activity against Gram-positive microorganisms such as the methicillin-resistant *Staphylococcus aureus* [2], which are common in lower respiratory tract infections. These characteristics have positioned vancomycin as one of the most suitable antibiotics for use in intensive care units (ICUs) [3].

Vancomycin is mainly administered intravenously, where its absorption in not required, and with a pharmacokinetic profile characterized by either a two- or three-compartment model. Based on different studies, and given its renal elimination, certain ranges have been established to study vancomycin’s behavior. For instance, in patients with preserved kidney function, the alpha distribution phase may last 30–60 min, while the half-life in the beta elimination phase varies from 6 to 12 h [4]. The binding of vancomycin to plasma proteins is variable (range of 10–82%), with an average of 50–55% [5], where its total volume distribution has been described in range of 0.4–1.0 L/kg. However, depending on the physiopathological condition of patients, these parameters can vary dramatically [6], affecting the efficacy of vancomycin.

Concerning the pharmacokinetic/pharmacodynamic (PK/PD) models, several studies have demonstrated that the ratio between the area under the 24 h concentration–time curve (AUC_0–24_) and the minimum inhibitory concentration (MIC) is the best predictor model for vancomycin activity [7]. However, this approximation does not consider the physiopathological changes in critically ill patients, where hypoalbuminemia, changes in volume distribution, renal dysfunction, and alterations in tissue penetration are frequent [8,9]. Moise-Broder et al. observed that AUC_0–24_/MIC values of ≥400 present successful outcomes in patients with *S. aureus*-associated pneumonia [10], while lower values are associated with a poor eradication of the infection, longer treatment duration, and high mortality rate [11]. The optimal serum vancomycin trough concentration has been defined as ≥10 mcg/mL, and 15–20 mcg/mL for pathogens with an MIC between 1 and 1.5 mcg/mL or complicated infections (endocarditis, osteomyelitis, meningitis, and nosocomial pneumonia) [12]. Plasma trough concentrations <10 mcg/mL are associated with resistance generation, while trough levels >20 mcg/mL have been linked with toxic effects, mainly nephrotoxicity [13,14].

In general, vancomycin administration is performed empirically and via intermittent infusions, as no clinical superiority has been demonstrated with prolonged or continuous infusion [15]. This therapeutic scheme assumes that optimum concentrations with an adequate AUC_0–24_ are obtained before the fourth or fifth dose (second or third day), which generally coincides with the equilibrium or steady state (SS) in patients with preserved renal function [16,17]. However, in critically ill patients, who may often have renal dysfunction, the vancomycin half-life increases, and the administration intervals must be modified, taking several days before SS is reached. This is a very delicate problem, because their critical conditions may be aggravated very rapidly without an adequate treatment. In this context, it has been reported that an initial vancomycin loading dose is useful to achieve adequate serum concentrations and an adequate AUC_0–24_ from the first day of treatment, thus avoiding the appearance of resistance or therapeutic failure and achieving a faster clinical response [18,19].

Therefore, in critically ill patients, it is crucial to adapt the vancomycin treatment to their special condition to obtain efficacy from day one, considering this individually for loading and maintenance dosing [20]. The aim of this study was to individualize a dosage regimen of vancomycin in a cohort of critically ill Chilean patients by using a population pharmacokinetic software with the aim of optimizing the pharmacological treatment, to offer greater therapeutic success and patient safety and minimize antibiotic resistance due to the selective pressure of susceptible microorganisms.

## 2. Results

### 2.1. Demographics and Baseline Characteristics

There were 173 patients admitted to the ICU, of which 51 met the inclusion criteria for this clinical study (29.5% of the total). Out of these 51, 36 were male and 15 were female, with an average age of 56.19 ± 14.16 years and with a stable estimated glomerular filtration rate (eGFR). With the Acute Physiology and Chronic Health Evaluation (APACHE) II classification system, the severity score was 21 in this group of patients, in a scale between 0 and 30. The most prevalent pathologies in patients were high blood pressure (23%), cancer (18%), and type-II diabetes mellitus (17%). All these characteristics are detailed in Table 1.

### 2.2. Vancomycin Treatment Characteristics

**Figure 1:**
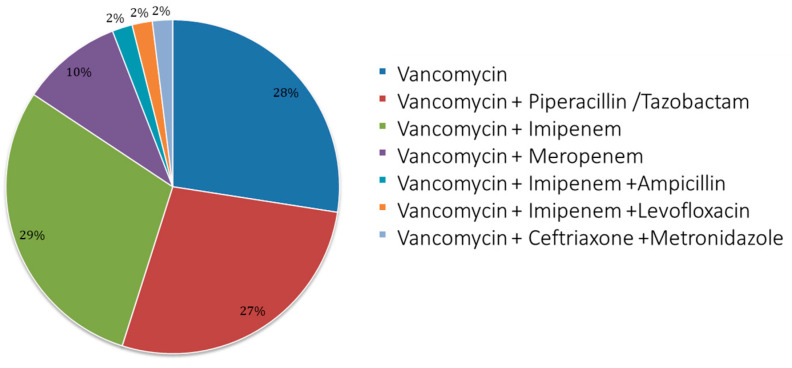
Antibiotic therapy scheme used. Pie chart for use of monotherapy and therapies associated with vancomycin (bi- and tri-therapy). The schemes for patients were the following: vancomycin = 14; vancomycin + piperacillin/tazobactam = 14; vancomycin + imipenem = 15; vancomycin + meropenem = 5; vancomycin + imipenem + ampicillin = 1; vancomycin + imipenem + levofloxacin = 1; vancomycin + ceftriaxone + metronidazole =1.

Among the sample, 31 patients started with an empirical therapy and 20 with targeted therapy. Figure 1 shows details of the vancomycin use and treatment scheme; mostly, patients received bi-therapy with imipenem (29%), followed by monotherapy (28%) and then tri-therapy associated with piperacillin/tazobactam (27%).

### 2.3. Loading and Maintenance Doses Analyzed by the Antibiotic Kinetics^®^ Software

**Figure 2:**
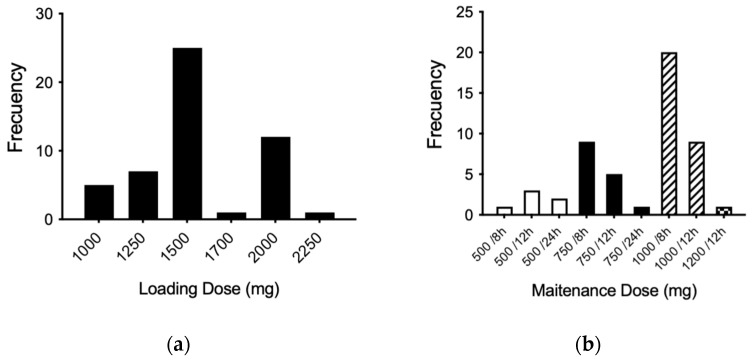
Frequencies for loading (**a**) and maintenance (**b**) doses (mg) according to the population model calculated. The maintenance frequency doses are expressed according to those temporarily used for each dose: 500 mg (white bars), 750 mg (black bars), 1000 mg (dashed line bars), and 1200 mg (black square bar).

According to the theoretical population pharmacokinetic model established by the Antibiotic Kinetics^®^ software (online version, RxKinetics), the most prescribed loading dose was 1500 mg, followed by 2000 mg (Figure 2a). In addition, the most widely used maintenance dose was 1000 mg every 8 h (three times a day), followed by the dose of 1000 mg every 12 h and the dose of 750 mg every 8 h (Figure 2b).

### 2.4. Analysis of Vancomycin Pharmacokinetics by Using the Antibiotic Kinetics^®^ Software

**Figure 3:**
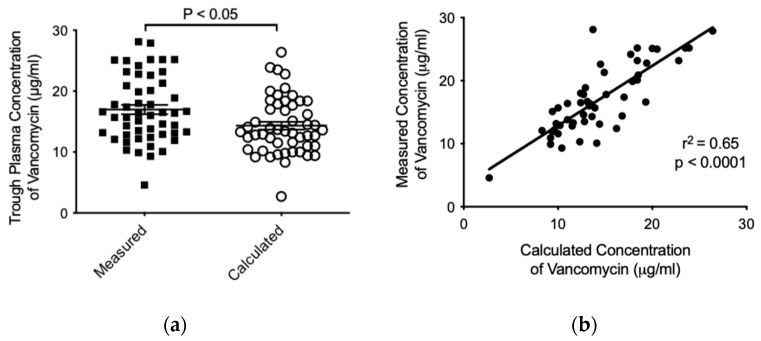
Analysis of vancomycin pharmacokinetics by using the Antibiotic Kinetics^®^ software. Mann–Whitney test comparison (**a**) and linear regression (**b**) among calculated and measured trough concentrations of vancomycin (μg/mL). In (**a**), data are presented as mean ± SD for each group (*n* = 51).

The average trough concentration measured for vancomycin in patients was higher in comparison to the concentration calculated by the Antibiotic Kinetics^®^ software (16.98 ± 5.423 versus 14.33 ± 4.630 μg/mL, respectively, *p* < 0.05) (Figure 3a). Finally, and with the aim to determine whether there was a relationship among calculated and measured trough concentrations of vancomycin, we studied both variables and found a significant positive correlation (r^2^ = 0.65; *p* < 0.0001) (Figure 3b). Importantly, no vancomycin-associated adverse effect was observed in the patients during the treatment.

## 3. Discussion

Vancomycin is one of the most commonly used antibiotics in health systems worldwide, which represents a challenge of its blood concentration monitoring and continuous revisions of its different intravenous administration strategies in critically ill patients. This calls for efforts to achieve a more efficient clinical response by reaching therapeutic concentrations as soon as possible, as well as avoiding the appearance of resistance and avoiding therapeutic failure [21]. Such practices impact cost-effectiveness, which supports improved management processes and optimization of resources [22].

Vancomycin treatment is widely used in many patients with impaired renal function and, in these cases, the plasma trough levels of the drug should be carefully measured for each patient. That avoids toxicity, obtaining subtherapeutic plasma concentrations and avoiding further resistance to glycopeptide antibiotics [23,24].

In this study, the majority of the individuals we could sample were male (70.6%); to contextualize this great disparity in gender, the Department of Statistics of our center was consulted for a list of admissions and discharges during the study period, and it was observed that there were a greater number of male patients (60.1%), thus our sample was nearly representative of the admissions in this period. Our center has a high-complexity Maternity and Gynecology Unit that cares for much of the population of Santiago, with many of these patients admitted to the intensive care unit due to gynecological complications during pregnancy. Considering that one of the exclusion criteria in this study was pregnancy in women, that could explain this gender disparity. In addition, this specific group had large changes in volumes of distribution and purification clearance, among other variations [25,26].

Concerning the potential adverse drug interactions that may arise from the use of vancomycin, it is important to note that it is not subject to hepatic metabolism and is primarily eliminated unchanged by the kidneys [27]. Due to its pharmacokinetic profile, vancomycin does not typically engage in metabolic drug–drug interactions. Additionally, the medications used in our study—piperacillin/tazobactam, imipenem, meropenem, ampicillin, levofloxacin, ceftriaxone, and metronidazole—do not share significant metabolic pathways with vancomycin, nor are they known to alter its pharmacokinetics in a clinically relevant manner. Therefore, these agents may be co-administered in clinical practice as part of empirical or targeted therapy, and their concurrent use with vancomycin is not expected to result in adverse drug interactions related to metabolism. The only potentially relevant interaction may occur with piperacillin/tazobactam, which has been associated with an increased risk of renal toxicity when administered concurrently with vancomycin [28], though this was not an effect we observed.

From Figure 3, it can be seen that the trough values of plasma vancomycin concentrations predicted through the theoretical population model Antibiotic Kinetics^®^ are approximately within the range of safety for patients [29,30]. The trough concentrations calculated by this model are generally lower when compared with trough values of measured serum vancomycin concentrations, considering the total population of patients monitored during the period covered by this study. However, the population pharmacokinetic model has been considered as an efficient predictor of serum vancomycin concentrations measured by the immunological method [18,22]. Critically ill patients routinely given vancomycin doses often reach concentrations outside the suggested therapeutic range for treatment of serious infectious diseases [8,31,32]. In our experience, we also observed patients with a measured plasma trough concentration >20 mcg/mL, with any adverse drug reaction detected.

In addition, pharmacokinetic variables in critically ill patients of extreme ages, with a special physiopathological condition, sex, weight, height, etc., are subject to higher inter-variability [33,34]. For example, the volume distribution and clearance elimination for vancomycin are altered in overweight patients, which conditions the dose adjustment in this group of patients [35]. However, these variables can be adjusted by the Antibiotic Kinetics^®^ program, and this adjustment was not considered because the new pharmacokinetic variables had operator-dependent values, generating a possible bias when making comparisons between measured and calculated values.

In this study, it was decided to assume standard pharmacokinetic variables for all patients studied [36]. However, our results guide the use of individualized doses for each patient, rather than the dose usually used for all adult subjects. In addition, the patient’s characteristics (age, weight, height, underlying pathologies, etc.) and other pharmacokinetic variables that may alter the plasma concentrations of vancomycin required to treat serious infections should be considered depending on the site of action [37]. This can be explained by the wide range of plasma protein binding that vancomycin possesses, according to the nutritional status and degree of renal dysfunction that the individual may present. However, critically ill patients normally present hypoalbuminemia after the initial reanimation with volume [38]. In this context, it is important to note that vancomycin binding to albumin or other plasma proteins is less than 50% in critically ill patients [39,40]. Therefore, the variability in protein binding of vancomycin, or its free fraction in the blood, should not be the first variable to be considered in this disease status. Nevertheless, it is important to evaluate this aspect in different studies, to improve our understanding of pharmacokinetic and pharmacodynamic aspects of vancomycin in critically ill patients.

In addition, it should be considered that the volume of distribution used when predicting a theoretical trough level significantly impacts the result of the real value obtained in the blood [35]. This is explained by the wide distribution of vancomycin, depending on anthropometric characteristics, severity of condition, and renal function of the patient. It is also important to consider that a single-compartment model is assumed [41]. Despite this, having used higher doses than those usually administered, no patient presented or manifested any adverse event associated with the vancomycin dosage, and all the subjects studied were able to complete their antibacterial treatment satisfactorily, supporting the experimental strategy used to ensure patient safety. In general, monitoring of plasma concentrations in critical patients is essential, considering the serious adverse events associated with and described for vancomycin usage (deterioration of kidney function, among others).

Currently, various guidelines recommend achieving AUC_0–24_/MIC values between 400 and 600 as a safety and efficacy target for methicillin-resistant *S. aureus* infections [17]. In clinical practice, monitoring vancomycin trough plasma concentrations can help ensure appropriate therapeutic outcomes and enhance patient safety in the individualized management of critically ill patients. Despite the limited number of patients included in this study, we suggest that it is crucial to consider additional variables—such as pathophysiology, comorbidities, infection site, and pharmacokinetic variability—which play a key role in accurate dosing. Typically, vancomycin dosing and regimens are applied uniformly across patients. However, we emphasize the need for personalized treatment strategies to optimize therapeutic responses in critically ill patients.

## 4. Materials and Methods

### 4.1. Subjects and Patient Selection

Patient selection was carried out at the ICU of the Hospital Clínico San Borja Arriarán, an adult tertiary hospital, between May and December 2015. The research was authorized by the Ethics Committees of the University of Chile, Faculty of Medicine (resolution on 11/08/2015, project N°033-2015, Acta AP-73) and the Central Metropolitan Health Service (resolution on 20/05/2015), in accordance with the procedures suggested in the Declaration of Helsinki, and according to Chilean Laws 20.120, 20.584, and 19.628 and the guidelines for Good Clinical Practices. All the patients included in this study, or their relatives, underwent an informed consent process and signed an informed consent document approved by the Ethics Committees.

We included patients with severe sepsis receiving empirical or directed treatment with intravenous vancomycin, according to the physician prescription, and patients in whom adequate measurement of plasma trough levels could be performed. On the other hand, we excluded pregnant women and patients on renal replacement therapy or in stage 5 for chronic kidney disease.

### 4.2. Vancomycin Loading Dose and Patient Follow-Up

Vancomycin intravenous treatment was initiated according to the clinical conditions of patients and in schemes of monotherapy or combined with other antimicrobials (bi- or tri-therapy), which was indicated by the treating physician and validated by the infectious disease team. The criteria of vancomycin use involved an empirical therapy or a targeted therapy in patients with isolated microorganisms and sensitivity to vancomycin treatment. The latter group also included patients with concomitant infection or those who started targeted therapy after the empirical one.

The theoretical calculation of vancomycin loading and maintenance doses was carried out using the Antibiotic Kinetics^®^ software (online version, RxKinetics) [42], a population pharmacokinetic technique, which uses anthropometric variables and laboratory analyses suggesting a dose regimen for a specific antibiotic through population variables. For vancomycin, a Bayesian mono-compartmental model was proposed after monitoring the drug plasma trough concentration and adjusting the further antibiotic treatment [32,37,43].

It is important to note that in the ICU, strict, permanent monitoring is performed of all clinical and laboratory parameters (renal, hepatic, cardiac, respiratory, metabolic function, etc.). Therefore, and because the potential risk of vancomycin-induced nephrotoxicity, continuous monitoring of the eGFR was performed for each patient using the Cockcroft–Gault (CG) equation, as well as the 6-variable Modification of Diet in Renal Disease (MDRD-6v) formula. To minimize adverse drug reactions, an active pharmacovigilance system was utilized by pharmacists in the ICU [44].

### 4.3. Vancomycin Plasma Concentration Assay

After the loading dose and during the subsequent maintenance doses of vancomycin, blood samples were obtained. The collections were performed 30 min before the next dose (trough level), and plasma trough levels of vancomycin were measured by the ADVIA Centaur^®^ CP immunoassay system (Siemens Healthineers, Erlangen, Germany).

### 4.4. Data and Statistical Analysis

Correlation data among calculated versus measured trough concentrations for vancomycin were analyzed using Graph Prim 5.0f, and their difference was tested using the Mann–Whitney nonparametric test. A *p*-value < 0.05 was considered statistically significant. All analyses were performed using GraphPad Prism version 10.0.

## 5. Conclusions

In this study, a vancomycin dosage regimen was successfully introduced on an individual basis for each critically ill patient within the usual clinical practice by the treating physician. The introduction of this dosage regimen for vancomycin ensured its efficiency and safety, reducing the possibility of generating in-hospital resistance due to antibiotic pressure and reducing the risk of therapeutic failure due to inadequate doses. No risks or adverse events occurred during the treatment associated with this practice, and therapeutic effectiveness was achieved with vancomycin through a population-based pharmacokinetic model, considering the conventional procedure of the treating physician setting the dosage.

## Figures and Tables

**Table 1:** Demographics and clinical characteristics.

**Characteristics**	**Baseline Values (*n* = 51)**
Age (years)	58 (18–79)
Gender (M/F)	36/15
Height (m)	1.68 (1.43–1.85)
Weight (kg)	75 (45–105)
Serum creatinine (mg/dL)	0.80 (0.28–2.07)
eGFR (mL/min/1.73 m^2^) _CG equation_	103 (20–208)
eGFR (mL/min/1.73 m^2^) _MDRD-6v formula_	84 (19–151)
APACHE II score	21 (15–38)
Comorbidities	
Hypertension	23%
Cancer	18%
Type II diabetes mellitus	17%
Non-hypertensive cardiomyopathies	11%
Human immunodeficiency virus	9%
Obstructive pulmonary syndrome	8%
Dyslipidemia	6%
Tuberculosis	4%
Hepatitis	2%
Others	2%

Demographics and clinical characteristics (*n* = 51 patients). Data are presented as medians for continuous variables and as numbers or % for discontinuous variables. eGFR; estimated glomerular filtration rate, CG; Cockcroft–Gault equation, MDRD-6v; 6-Variable Modification of Diet in Renal Disease; APACHE; Acute Physiology and Chronic Health Evaluation.

## Data Availability

All the data produced or examined in this study are available within the article and its supplementary online materials. For additional information, please contact the corresponding author.

## Abbreviations

The following abbreviations are used in this manuscript:

APACHE	Acute Physiology and Chronic Health Evaluation
AUC_0–24_	Area under the 24 h concentration–time curve
CG	Cockcroft–Gault
eGFR	Estimated glomerular filtration rate
ICU	Intensive care unit
MDRD-6v	6-Variable Modification of Diet in Renal Disease
MIC	Minimum inhibitory concentration
PK/PD	Pharmacokinetic/pharmacodynamic
SS	Steady state
